# Tuberculosis infection control measures in health care facilities offering tb services in Ikeja local government area, Lagos, South West, Nigeria

**DOI:** 10.1186/s12879-016-1453-y

**Published:** 2016-03-15

**Authors:** Y. A. Kuyinu, A. S. Mohammed, O. O. Adeyeye, B. A. Odugbemi, O. O. Goodman, O. O. Odusanya

**Affiliations:** Department of Community Health and Primary Health Care, Lagos State University Teaching Hospital, Ikeja, Nigeria; Department of Medicine, Lagos State University Teaching Hospital, Ikeja, Nigeria

**Keywords:** TB infection control, Health care workers, Health facilities, Barriers

## Abstract

**Background:**

Tuberculosis infection among health care workers is capable of worsening the existing health human resource problems of low - and middle-income countries. Tuberculosis infection control is often weakly implemented in these parts of the world therefore, understanding the reasons for poor implementation of tuberculosis infection control guidelines are important. This study was aimed at assessing tuberculosis infection control practices and barriers to its implementation in Ikeja, Nigeria.

**Methods:**

A cross-sectional study in 20 tuberculosis care facilities (16 public and 4 private) in Ikeja, Lagos was conducted. The study included a facility survey to assess the availability of tuberculosis infection control guidelines, the adequacy of facilities to prevent transmission of tuberculosis and observations of practices to assess the implementation of tuberculosis infection control guidelines. Four focus group discussions were carried out to highlight HCWs’ perceptions on tuberculosis infection control guidelines and barriers to its implementation.

**Results:**

The observational study showed that none of the clinics had a tuberculosis infection control plan. No clinic was consistently screening patients for cough. Twelve facilities (60 %) consistently provided masks to patients who were coughing. Ventilation in the waiting areas was assessed to be adequate in 60 % of the clinics while four clinics (20 %) possessed N-95 respirators. Findings from the focus group discussions showed weak managerial support, poor funding, under-staffing, lack of space and not wanting to be seen as stigmatizing against tuberculosis patients as barriers that hindered the implementation of TB infection control measures.

**Conclusion:**

Tuberculosis infection control measures were not adequately implemented in health facilities in Ikeja, Nigeria. A multi-pronged approach is required to address the identified barriers to the implementation of tuberculosis infection control guidelines.

## Background

The control of tuberculosis (TB) continues to be important because of the ease of spread due to its transmission by airborne droplets; and the increasing mortality and morbidity from the disease. At onset, the symptoms of the disease are non-specific, thus, they tend to be overlooked until the disease has progressed. This may result in increased risk of transmission among members of the community. In addition to this, the association between tuberculosis and human immunodeficiency virus (HIV), together with the increasing prevalence of multi drug resistant-tuberculosis (MDR-TB) and extensively drug resistant-tuberculosis (XDR-TB) make TB control challenging [1, 2]. Within health facility settings, the risk of TB infection and disease is increased by the interaction between patients with active TB and other patients or health care workers (HCWs) in clinics and hospitals where tuberculosis infection control (TBIC) measures are lacking [3, 4]. The risk is worse for patients or HCWs with HIV infection [5].

A systematic review of TB infection among HCWs in low - and middle-income countries reported a 54 % prevalence (range 33 % to 79 %) of latent tuberculosis infection (LTBI), an annual incidence of 69 to 5,780/100,000 population and attributable risk for TB disease ranging from 25 to 5, 361/100,000/year. The risk for acquiring TB was found to be higher among HCWs in in-patient TB facilities, patient attendants, nurses and clinical officers [6]. A study in south India reported a 47.5 % prevalence rate of LTBI amongst young nursing trainees. The skin test positivity was strongly associated with time spent in health care after adjusting for age at entry into health care work [7]. In Turkey, HCWs had a five-fold higher incidence of TB (RR 4.9), this being higher in nurses (RR 6.7) compared to the general population [8]. It is clear that HCWs are at a higher risk for TB disease and should be adequately protected. Tuberculosis disease among HCWs renders them too ill to work and is therefore capable of worsening the existing health human resource problems of low- and middle- income countries. Implementation of TBIC in developing countries where TB burden is usually high tends to be inadequate. This was reported in a study from South Africa in which the incidence of smear positive tuberculosis was twice as high among staff of a TB treatment centre than in the general population [9].

A TBIC plan for health facilities provides a framework to prevent the spread of infection in the work place. The World Health Organization (WHO) recommends four categories of TBIC measures in health facilities that care for TB suspects or cases [1]. These measures are: managerial, administrative, environmental and respiratory protective measures, which when well applied have been found to minimize TB transmission in health facilities. The guidelines include availability of a TBIC plan, HCWs training, prompt identification of TB suspects, triage, patient education about TB, sputum collection practices (in a well ventilated area), well ventilated facilities and use of protective wear [1]. These measures have been found to be effective in the control of nosocomial transmission of multidrug resistant tuberculosis (MDR-TB) [10–12]. For example, a study in Miami, Florida, United States of America (USA) amongst patients infected with the human immune-deficiency virus found that exposure to MDR-TB was reduced from 80 to 0 % and skin-test conversion among HCWs on the HIV ward dropped from 28 to 0 % following implementation of these measures [10]. Another study in a teaching hospital in New York City found very similar findings [11].

Furthermore, HCWs often lack adequate knowledge about TB and infection control and as such are prone to nosocomial infections including tuberculosis [13, 14]. Therefore, it is important to study TBIC measures and barriers to its implementation in facilities providing care for TB patients especially in Nigeria which has a disproportionate burden of TB and HIV with corresponding high levels of morbidity and mortality attributable to both diseases [15]. In 2013, in Nigeria, the prevalence of TB and HIV per 100,000 population were 161 and 2030 respectively [16]. The aim of this study was to assess the level of implementation of TBIC measures in all health facilities caring for TB suspects or cases and to determine HCWs’ perceptions of barriers to implementation of TBIC in Ikeja Local Government Area (LGA), Lagos State, southwest, Nigeria.

## Methods

### Background information to study area

Ikeja Local Government Area is one of the 16 urban LGAs in Lagos State. There are 20 (sixteen government owned of which one is a referral centre and four privately owned) TB care facilities, in the LGA. The centres provide sputum examination for diagnosis, registration of diagnosed cases, free treatment and follow-up of TB patients. The State Tuberculosis, Leprosy and Buruli Ulcer Control Programme provides supervision for the TB directly observed treatment short-course (DOTS) services in these facilities to ensure the availability and quality of the free treatment and sputum examination provided in these centres. In each health centre, a nurse or community health officer is usually responsible for the TB patient care. All the facilities use the same protocol for TB care. None of the facilities was purposely built for TB control. Only 4 (20 %) facilities had capability for sputum smear for TB diagnosis.

### Study design

A cross-sectional study was conducted at TB care facilities in Ikeja LGA, Lagos State using both quantitative and qualitative data collection methods to assess the availability and implementation of TBIC practices. The quantitative aspect included a health facility survey questionnaire and observation of TB prevention practices. The qualitative aspect was done using focus group discussions (FGDs) with HCWs. The FGDs attempted to elicit in-depth understanding of those factors influencing implementation of TBIC measures.

### Study duration

The total duration of study was five months (March- July 2014)

### Health facility survey

The facility survey questionnaire was developed from the 2009 WHO guidelines for TBIC in health care facilities and assessed the adequacy of structural, administrative and environmental control measures in the facilities [1]. This was done through interviews of the facility managers (and in their absence, the TB/HIV focal persons, the officer in-charge of the TB clinic or the Infection Control Officer) and by observation of TBIC practices.

Data was collected on the adequacy of ventilation adequacy at the waiting areas and consulting rooms through measuring the length and width of the windows and the floor space. The window (W) area was calculated by multiplying the height of the window with the width. The area of all the numbers of windows in each patient-interaction space (waiting area and consulting room) was summed up to give the total window area (ARW). The floor area (ARM) was calculated by multiplying the room length with the width. The ratio of the total window area (ARW) to the floor area (ARM) was used to assess adequacy of air ventilation. Ventilation = ARW/ARM × 100 %.

Ventilation was considered adequate if the ratio was ≥ 20 %. Ventilation in the outpatient ward in terms of flow of air was considered adequate if there were windows of equal size on the two opposite sides of the room, and ARW was ≥ 20 %. Ventilation in the consultation room in terms of flow of air was considered adequate if there were windows of equal size on the two opposite sides of the room and ARW was ≥ 20 %. The formula was adapted from the tool described in the appendix for the Ugandan National TBIC guidelines [17], and has been used in a previous study [4]. It has not been validated to measure actual air exchange.

### Non-participant observations of TB infection control practices

An unannounced direct observation of control measures was carried out once a week over a three week period in each health facility. This was done in order to provide convergent validity to the responses from the managers. Using an observation checklist, data was collected on patient screening for cough, where the screening was done, availability of masks for patients with cough and whether TB suspects had a separate waiting area. The observations were recorded as ‘always’ if the practice was observed on all the three days of observation, ‘occasionally’ if the practice was not consistently observed or ‘not at all’ if the practice was not seen.

### Focus group discussions

Four FGDs were conducted with HCWs (different from those who participated in the survey) in the health facility with the use of an interview guide to explore their experiences and perceived challenges in implementing the TBIC measures. The FGD participants were purposively recruited with the help of the facility managers who had introduced the researcher to the participants and the objective of the discussion stated before its commencement. The researchers had no prior relationship with the participants. All the discussions were face to face and lasted between one to two hours. The participants and researchers were the only ones present during the discussion. The audio recording of the FGDs was done while notes were taken during the discussions. Each FGD group comprised of 10 participants and no repeat discussion was carried out with any group of participants.

### Data analysis

Quantitative data from the facility survey questionnaire and facility observation checklists were entered into SPSS version 21, cleaned and analysed. Uni-variate analysis was done using frequencies and percentages. The FGDs were transcribed verbatim. Transcripts were read several times to get an overall picture of the contents and common themes were developed from the data. The authors reviewed themes for agreement. The qualitative finding was used to complement findings from the facility survey (questionnaire and observation) data.

### Ethical considerations

Ethical approval for the study was obtained from the Lagos State University Teaching Hospital Health Research and Ethics Committee. Informed written and verbal consent was obtained from participants at the time of data collection (for the surveys and FGDs respectively). Confidentiality and use of data for research purposes was maintained throughout the study.

## Results

### Managerial and administrative control measures

As shown on Table 1, none of the facilities had a written TBIC plan. In addition no facility risk assessment had been conducted for any of the DOTS centers. Only 30 % of the facilities reported having a dedicated person/committee responsible for TBIC. None of the facilities practiced the screening of patients for cough on arrival at the hospital. More than half (60 %) of the DOTS clinics reported providing face masks for patients with cough. However, on direct observation, only 20 % of the clinics consistently ensured that patients who were coughing wore face masks. Furthermore, non-touch waste disposal for used mask or tissue paper was only available in seven (35 %) of the health facilities.

**Table 1:** Managerial and Administrative Control Measures for Tuberculosis Infection in TB Facilities in Ikeja LGA

Control Measures^a^	Frequency (*n* = 20)	Percentage
Availability of written TB infection control plan	0	0
Availability of a dedicated Person/Committee for infection control	6	30
Conduct of risk assessment in facility	0	0
Screening of patients screened for cough on arrival at facility	0	0
Provision of face mask for patients coughing	12	60
Availability of non-touch waste disposal	7	35
Provision of health education on cough hygiene	19	95
Display of posters on cough etiquettes in facility	9	45
Segregation of TB cases from others	13	65
Confidential TB screening for staff	3	15
Staff training on TB infection control	2	10
Availability of N-95 respirators in health facilities	4	20

^a^Positive responses only

All the clinics with the exception of one (95 %) reported that they provided patients with health education on cough hygiene but only 45 % had posters on cough etiquettes displayed. Moreover, direct observation revealed that consistent delivery of health education on cough etiquette was given in only 20 % of the clinics and 55 % of the clinics gave health education occasionally. The low rate of screening for cough amongst patients was consistent with the findings from direct observation where majority of clinics (90 %) did not screen patients for cough before they entered enclosed areas. Most of the observed infection control practices were not performed all the time and were lower than the rates reported by the HCWs (Table 2). The implementation of these measures and observations were similar in public and private health facilities.

**Table 2:** Direct Observation of Tuberculosis Infection Control Practice in DOTS Clinics of Ikeja LGA

Practices observed	Frequency (*n* = 20)	Percentage (%)
Screening for cough before patient enters enclosed area:		
Always	0	0
Occasionally	2	10
Not at all	18	90
Use of face mask by patients:		
Always	4	20
Occasionally	6	30
Not at all	10	50
Health education on cough etiquette given:		
Always	4	20
Occasionally	11	55
Not at all	5	25
Sputum production done in separate well-ventilated area:		
Always	0	0
Occasionally	0	0
Not at all	20	100
Staff use of N-95 respirators:		
Always	0	0
Occasionally	1	5
Not at all	19	95

### Environmental control measures and respiratory protection

All the clinics used a mixture of mechanical (electric fans) and natural ventilation (opened windows). Three quarters of the clinics had cross ventilation in the patient waiting areas while only 35 % had cross ventilation in the consulting rooms. In most of the centres, patients were given cups to produce sputum outside the clinic areas unsupervised.

Only 10 % of the clinics had designated sputum collection areas (no patient was seen producing sputum in the designated areas during observations) and none of them used ultraviolet germicidal irradiation for sterilization of the air.

The window to floor area ratio was adequate for more than half of clinics’ patient waiting areas (60 %) while it was only adequate for half of the consulting rooms (see Table 3).

**Table 3:** Environmental Control Measures for Tuberculosis Infection in DOTS Healthcare Facilities in Ikeja LGA

Control Measure^a^	Frequency (*n* = 20)	Percentage (%)
Ventilation System:		
Mixed (both natural and mechanical)	20	100
Cross Ventilation in Waiting Area	15	75
Cross Ventilation in Consulting Room	7	35
Use of designated area for sputum Collection	0	00
Use of ultraviolet germicidal irradiation	0	0
Adequacy of window/Floor Area Ratio in waiting area (≥20 %)	12	60
Adequacy of window/Floor Area Ratio in consulting room (≥20 %)	10	50

^a^Positive responses only

### Obstacles to implementation of tuberculosis infection control

The HCWs identified several obstacles precluding the proper implementation of TBIC within their facilities. These obstacles included weak managerial support, poor funding, structural inadequacy, shortage of manpower, stigmatization and poor compliance by patients.

### Weak managerial support

HCWs perceived that managerial support was weak or lacking in most of the clinics. The DOTS clinics were perceived to be a low priority for the management of the health facilities.

‘…when it comes to matters of our clinic the management does not take it seriously…’

In the referral hospital it was reported that the management was not supportive because the clinic was not generating income when compared to other departments.

‘… the management does not take us seriously because the clinic does not make money for the hospital like other clinics. For example is this how they treat the theatre?…’

### Poor funding

HCWs also complained of poor funding as an impediment to proper implementation of TBIC. The recurring complaint was that there was almost non-existent funding of activities in the TB clinics from hospital management and limited funds from a few development partners. In one of the clinics, the management was said to refer request for funding back to development partners.

‘… whenever we ask for money to buy anything they tell us that the WHO should provide money …’

‘… they tell us not to collect any money from patients so anytime money is provided for buying face masks we give the patients, but most of the time there is no money…’

In the referral hospital, HCWs reported that the hospital management only spends money on the clinic when there is some benefit coming to the hospital from external bodies.

‘… We have been requesting for fans in our consulting rooms but the complaint was always that there was no money, but when the National Postgraduate Medical College of Nigeria was to accredit the hospital, the money was quickly provided. Similarly, when the National TB Control Programme wanted to provide Gene Xpert machines for the hospital, the management provided money for renovation of the clinic …’

### Structural inadequacy

Structural inadequacies were identified by HCWs as impediments to proper implementation of TBIC measures. Most clinics were housed in small buildings that did not provide enough space for segregating TB suspects/cases from other patients.

‘… yes it is good to separate TB patients from other patients, but how can we practice that here? Can’t you see how small and tight the place is? It is just not possible to start separating anybody …’

In one facility, HCWs complained that they were using a store as the DOTS clinic even though it had only one small window.

‘… when we wanted to start the clinic they moved us to a store with only one small window, so you see that ventilation cannot be enough …’

### Shortage of manpower

Another challenge to TBIC was shortage of manpower. Clinics were perceived as being under staffed and hence implementation of infection control measures was seen as an added responsibility that was difficult to carry out.

‘… We cannot afford to be giving health education all the time because the work is much and yet we are few …’

‘… you mean someone should just sit down to be monitoring if patients are coughing, how is that possible, how many are we here? …’

### Stigmatization of TB patients

HCWs mentioned stigmatization against TB as a major hindrance to practicing TBIC measures like screening for TB and segregating of TB suspects from other patients.

‘… it is really not possible to start separating people from others because they are coughing. There is already too much stigmatization, so this form of separation will only make things worse …’

### Poor compliance with instructions by patients

It was mentioned that there was difficulty in enforcing compliance with cough etiquettes by patients.

‘… we keep on telling the patients to cover their mouths when coughing, but they just won’t comply …’

## Discussion

Vital information regarding the level of TBIC implementation in Ikeja, Nigeria has been brought to light by this study. The good TBIC practices which the majority of health facilities reported were: providing health education on cough hygiene (95 %), segregation of TB cases from others (65 %) and provision of face masks to patients who are coughing (60 %).

However, using more objective methods it was observed that none of the clinics had a TBIC plan and that there was poor implementation of administrative measures like screening and separation of TB suspects/cases from other patients. In addition, HCWs were neither provided with confidential TB screening nor provided with training on TBIC. This finding is similar to what has been reported from Uganda [4].

Just like a similar study in Uganda, this study has established differences between self-reported and observed TBIC practices of HCWs [4]. Although 60 % and 95 % of the clinics reported the provision of face mask for coughing patients and education of patients on cough hygiene, respectively, only 20 % of them, were observed implementing both measures consistently. Furthermore, although 65 % of clinics reported segregation and fast-tracking of TB suspects/cases, none of them were observed doing it. This may be an indication that knowledge - practice gap about TBIC exists among HCWs. In addition, some of the failures may be due in part to shortage of manpower as suggested from the focus group discussions.

The concerns of the HCWs at the facilities (that they were not priority for the management) and the state of the infrastructural deficiencies at the clinics are similar to what has been reported from South Africa where up to 49 % of the staff felt that their hospitals did not care about them and were not working to prevent TB infection among staff [18]. In a large number of facilities surveyed (65 %) segregation of patients was done but sputum collection was not done in the segregated area in both private and public facilities. This may be due to the structural inadequacies and will further worsen the likely implementation of the environmental control measures.

The perceived barriers to implementation of TBIC in this study included weak managerial support, limited human resource, stigmatization against TB and lack of space. These findings are not different from what has been reported from Uganda and South Africa [4, 18]. In addition, the non-availability of masks in our study is similar to what was found among Ethiopian HCWs where only 8 % reported that face masks were regularly available and 76 % cited a lack of adequate infrastructure to isolate suspected/known TB patients [19]. The low implementation of TBIC observed in both public and private facilities may be largely due to structural inadequacies as none of the facilities are purpose built. In this study availability of N-95 masks was 20 % but was not used consistently in any, and occasionally used in only 5 % of the health facilities. This is an area of concern as the HCWs are not using even the small number of protective equipment provided. Ventilation was adjudged to be inadequate in consulting rooms and patient waiting areas. These deficiencies in the opinion of staff were due to weak managerial support and poor funding. Other researchers have also cited similar reasons for poor environmental control measures [4, 18]. The reasons for these inadequacies may not be obvious but could be due to limited resources and perhaps other competing conditions and over dependence on donor funds.

It is known that administrative control measures are the easiest to implement but this study has shown that TB care facilities in Ikeja, Lagos, Nigeria have not been able to implement them adequately. The poor level of implementation of these control measures may impact negatively on TB infection control in these facilities in many ways including increased exposure of HCWs to TB and the ease of spread of nosocomial infection in such facilities [7, 20]. These may then worsen the health manpower shortage in TB care where there is a known shortage of manpower and should not be allowed. Moreover, it is known that institutional administration support is critical in providing necessary resources for TBIC, organizing trainings and monitoring compliance with infection control guidelines [18, 19].

This study is limited to one local government area in Lagos State. A larger study involving all the LGAs in the state is therefore recommended in order to determine the extent of the implementation of TBIC in other health facilities in Lagos State. In addition, the knowledge of the HCWS at these facilities was not evaluated and the risk of staff contracting tuberculosis was not assessed. This study did not explore the impact of HIV on TBIC. It is an important area to explore in the future.

## Conclusion

The implementation of TBIC in the facilities in Ikeja, Lagos was poor. Weak managerial support, poor funding, lack of space and staff have been identified as barriers to implementation of TBIC. Practical and affordable measures to reduce the risk of TB among HCWs in developing countries include diagnosis and treatment of infectious TB patients, investigation of TB suspects as out patients, environmental controls, use of face masks, patient cough hygiene and screening of health staff are all well-known and easy to implement [21]. Even though some of these were not consistently practiced at the facilities in this study, we recommend that compliance with them be increased through training of staff and creation of institutional commitment to implementing them. Other measures include supervision, improved funding, surveillance for TB among staff and increasing number of staff available for TB care. Purpose built facilities with adequate structural designs should be built for TB care in the future.

## Abbreviations

ARM: The floor area
ARW: Total window area
DOTs: Directly observed treatment short- course
FGD: Focus group discussion
HCW: Health care worker
HCWs: Health care workers
HIV: Human immunodeficiency virus
LASUTH: Lagos State University Teaching Hospital
LGA: Local government area
LTBI: Latent tuberculosis infection
MDR-TB: Multi drug resistant – tuberculosis
SPSS: Statistical package for social sciences
TB: Tuberculosis
TBIC: Tuberculosis infection control
USA: Unites States of America
W: Window
WHO: World Health Organization
XDR-TB: Extensively drug resistant – tuberculosis
